# Implication of Free Fatty Acids in Thrombin Generation and Fibrinolysis in Vascular Inflammation in Zucker Rats and Evolution with Aging

**DOI:** 10.3389/fphys.2017.00949

**Published:** 2017-11-22

**Authors:** Jérémy Lagrange, Mélusine Didelot, Amel Mohamadi, Lucy A. Walton, Saartje Bloemen, Bas de Laat, Huguette Louis, Simon N. Thornton, Brian Derby, Michael J. Sherratt, Bruno Fève, Pascal Challande, Riaz Akhtar, J. Kennedy Cruickshank, Patrick Lacolley, Véronique Regnault

**Affiliations:** ^1^Institut National de la Santé et de la Recherche Médicale, UMR_S 1116, Vandœuvre-lès-Nancy, France; ^2^Faculté de Médecine, Université de Lorraine, Nancy, France; ^3^Center for Thrombosis and Hemostasis, University Medical Center Mainz, Mainz, Germany; ^4^Faculty of Medical and Human Sciences, Institute of Cardiovascular Sciences, University of Manchester, Manchester, United Kingdom; ^5^Directorate of Radiography, School of Health Sciences, University of Salford, Salford, United Kingdom; ^6^Synapse Research Institute, Cardiovascular Research Institute Maastricht, Maastricht University Medical Center, Maastricht, Netherlands; ^7^School of Materials, University of Manchester, Manchester, United Kingdom; ^8^Faculty of Medical and Human Sciences, Institute of Inflammation and Repair, University of Manchester, Manchester, United Kingdom; ^9^Centre de Recherche Saint-Antoine Institut National de la Santé et de la Recherche Médicale-Université Pierre et Marie Curie, UMR_S 938, Paris, France; ^10^Institut Hospitalo-Universitaire ICAN, Paris, France; ^11^Assistance-Publique des Hôpitaux de Paris, Service d'Endocrinologie, Hôpital Saint-Antoine, Paris, France; ^12^UPMC, University of Paris, Paris, France; ^13^Centre National de la Recherche Scientifique, UMR 7190, Paris, France; ^14^Centre for Materials and Structures, School of Engineering, University of Liverpool, Liverpool, United Kingdom; ^15^Diabetes & Cardiovascular Medicine, Nutritional Sciences Division, King's College London, London, United Kingdom; ^16^CHRU Nancy, Vandœuvre-lès-Nancy, France

**Keywords:** vascular aging, blood coagulation test, obesity, fatty acids, thrombin generation

## Abstract

**Background:** The metabolic syndrome (MetS) and aging are associated with modifications in blood coagulation factors, vascular inflammation, and increased risk of thrombosis.

**Objectives:** Our aim was to determine concomitant changes in thrombin generation in the blood compartment and at the surface of vascular smooth muscle cells (VSMCs) and its interplay with adipokines, free fatty acids (FFA), and metalloproteinases (MMPs) in obese Zucker rats that share features of the human MetS.

**Methods:** Obese and age-matched lean Zucker rats were compared at 25 and 80 weeks of age. Thrombin generation was assessed by calibrated automated thrombography (CAT).

**Results:** Endogenous thrombin potential (ETP) was increased in obese rats independent of platelets and age. Clot half-lysis time was delayed with obesity and age. Interleukin (IL)-1β and IL-13 were increased with obesity and age respectively. Addition of exogenous fibrinogen, leptin, linoleic, or palmitic acid increased thrombin generation in plasma whereas adiponectin had an opposite effect. ETP was increased at the surface of VSMCs from obese rats and addition of exogenous palmitic acid further enhanced ETP values. Gelatinase activity was increased in aorta at both ages in obese rats and MMP-2 activity was increased in VSMCs from obese rats.

**Conclusions:** Our study demonstrated in MetS an early prothrombotic phenotype of the blood compartment reinforced by procoagulant properties of dedifferentiated and inflammatory VSMCs. Mechanisms involved (1) increased fibrinogen and impaired fibrinolysis and (2) increased saturated fatty acids responsible for additive procoagulant effects. Whether specifically targeting this hypercoagulability using direct thrombin inhibitors would improve outcome in MetS is worth investigating.

## Introduction

Atherothrombotic events and venous thromboembolism are associated with the metabolic syndrome (MetS), a cluster of risk factors for cardiovascular disease including insulin resistance (IR), abdominal adiposity, dyslipidemia, and hypertension (Dandona et al., 2005). Likewise, obesity is causally related to the high prevalence of MetS. Inflammation in MetS results in endothelial dysfunction and increased arterial stiffness (Weiss et al., 2013), probably through the action of matrix metalloproteinases (MMPs; Halcox et al., 2009). Aging is also associated with intimal thickening, breaks in the internal elastic lamina and impaired endothelial function leading to increased arterial stiffness (Wang et al., 1996).

A further cascade of obesity-induced chronic inflammation leads to increased tissue factor (TF; Samad et al., 2001) through the NF-κB pathway (Sonnenberg et al., 2004). Von Willebrand factor (VWF) participates in the prothrombotic state found in MetS (Lim et al., 2004). Total thrombin generation and platelet reactivity are increased in type 2 diabetes and older obese women (Beijers et al., 2010). Furthermore, as far as fibrinolysis is concerned, chronic inflammation, abdominal obesity, and IR all increase plasminogen activator inhibitor-1 (PAI-1) production, so reducing plasminogen conversion and leading to a hypofibrinolytic state (Alessi and Juhan-Vague, 2008; Suehiro et al., 2012).

Adipokine levels (adiponectin, leptin) as well as free fatty acid (FFA) metabolism are changed significantly in MetS (Matsuzawa et al., 2004; Wakil and Abu-Elheiga, 2009). Both are known also to be directly or indirectly implicated in haemostasis and increased thrombosis (Konstantinides et al., 2001; Restituto et al., 2010). Since haemostasis is modified in the MetS and during aging our hypothesis is that MetS, the related adipokines, and FFAs have a major impact on haemostasis changes, increased thrombotic risk and worsen the vascular phenotype. A major challenge is to elucidate the mechanisms leading to increased thrombosis during MetS and in the natural course of aging, and how they are related to the interaction between blood haemostasis and the vascular wall. Rodent models that mimic human MetS are major tools for understanding this pathophysiology (Sloboda et al., 2012).

Obese Zucker rats have a missense point mutation (fa/fa) in the leptin receptor gene that leads to hyperphagia and marked obesity (Phillips et al., 1996). These rats display also many other aspects of the human condition, such as IR, hypertension, and increased plasma lipid levels. We have shown previously that obese Zucker rats exhibited an increased age-dependent arterial stiffening which was greater in obese than lean, as well as endothelial dysfunction with increased systemic oxidative stress (Sloboda et al., 2012).

We have developed therefore a strategy combining “adult” (25-week-old) and “old” (80-week-old) Zucker rats with MetS characteristics and their lean controls and a vascular smooth muscle cell (VSMC) approach to investigate the role of FFAs and vascular inflammation in the prothrombotic properties of MetS. We first explored thrombin generation and its functional consequences on the fibrin network and on fibrinolysis in the blood compartment. To get insights into the underlying mechanisms we then examined thrombin generation at the surface of Zucker rat VSMCs and their MMP activity. We demonstrated that obesity from at least 25 weeks triggers increased thrombin generation in the blood compartment and at the surface of VSMCs via increased FFAs and associated vascular inflammation.

## Materials and methods

### Animals

Male Zucker rats with the MetS (MSZR, fa/fa; *n* = 18) and their age-matched male lean Zucker rat controls (LZR, FA/-; *n* = 18) were obtained from the breeding colony (animal facility, Faculty of Medicine, University of Lorraine, France). The animals were maintained at a constant temperature of 22–24°C, with a 12 h light-dark cycle (light beginning at 8 a.m.) and given free access to water and standard chow (A04, Scientific Animal Food and Engineering advance, Augy, France). The metabolic status of MSZR and LZR has been published previously (Sloboda et al., 2012).

Eighty weeks of age corresponds to 5 weeks before the mean maximum life span of rats from our local breeding colony.

This study was carried out in accordance with recommendations of the Animal Ethics Committee of the Institut National de la Santé et de la Recherche Médicale and conformed to the Guide for the Care and Use of Laboratory Animals, published by the National Institutes of Health. The protocols were approved by the Animal Ethics Committee of the Institut National de la Santé et de la Recherche Médicale.

### Blood sampling

Rats were anesthetized with isoflurane and whole blood was collected via a carotid catheter into syringes containing one-tenth the volume of 0.106 M sodium citrate. Platelet count was determined with an automatic cell counter (Micros 60 ABX model, Montpellier, France). Blood was centrifuged at 190 g for 10 min at room temperature to obtain platelet-rich plasma (PRP) and then at 1,750 g for 10 min to obtain platelet-poor plasma. PRP was adjusted to 200 × 10^9^ platelets/l by addition of autologous platelet-poor plasma and used for platelet aggregation and thrombin generation. Platelet-free plasma (PFP) was obtained by centrifugation of platelet-poor plasma at 13,000 g for 30 min at 4°C, and frozen at −80°C.

### Preparation of arterial cryo-sections

Artery cryo-sections were collected in the cross-sectional orientation and used subsequently for *in situ* gelatin zymography. The descending thoracic aorta was embedded in Optimal Cutting Temperature (OCT) medium and frozen using iso-pentane pre-cooled in liquid N_2_ and stored at −80°C until cryo-sectioning. Cryo-sections were cut at a thickness of 5 μm and mounted onto glass slides (Leica, Milton Keynes, UK) and stored at −80°C until use.

### Cell culture

The descending thoracic aorta was excised from rats after isoflurane anesthesia (4.5% in 1.5 l/min dioxygen) and exsanguination. VSMCs were isolated as described previously (Ait Aissa et al., 2015). VSMCs were grown in DMEM/F12 supplemented with 10% fetal bovine serum (Lonza, Basel, Switzerland). For thrombin generation assays, VSMCs at passages 3–5 were seeded (7,500 cells/well) in 96-well tissue culture flat-bottom plates (MICROTEST™96), grown to subconfluence and washed with HBS before use.

### Platelet aggregation

Blood was centrifuged at 190 g for 4 min followed by 70 s at 1,900 g at room temperature to obtain PRP and then platelets were sedimented by centrifugation at 5,000 g for 4 min. Platelets were re-suspended in Tyrode buffer (5 mM Hepes, 137 mM NaCl, 2.7 mM KCl, 12 mM NaHCO_3_, 0.4 mM NaH_2_PO_4_, 2 mM CaCl_2_, 1 mM MgCl_2_, 5.5 mM glucose, pH 7.3). Platelet aggregation was measured by turbidimetry at 37°C under stirred conditions. PRP or washed platelets were adjusted to 200 × 10^9^ platelets/l and were stimulated by 5 μg/ml collagen or 5 μM ADP (SD Innovation, Frouard, France). Aggregation was followed for 10 min using a TA-8V aggregometer (SD Innovation).

### Thrombin generation assay

Calibrated automated thrombinography (CAT) in PRP or PFP was performed in a microtiter plate fluorometer (Fluoroskan Ascent, ThermoLabsystems, Helsinki, Finland) using a dedicated software program (Thrombinoscope BV, Maastricht, The Netherlands) as reported previously (Regnault et al., 2004). All reagents were used at half the ordinary volume as follows: 40 μl PRP or PFP, 10 μl of 5 pM recombinant human tissue factor (TF) (Dade Behring, Marburg, Germany) and phospholipid vesicles (PV) consisted of phosphatidylcholine-serine-ethanolamine (PC/PS/PE) 60/20/20 mole% at a final concentration of 4 μM equivalent PS, 10 μl fluorogenic substrate and calcium. PV were replaced by buffer in PRP and VSMC experiments. Round-bottom 96-well Greiner blue plates were used for PFP and PRP, and MICROTEST™96 plates for VSMC monolayers. Thrombin generation curves were recorded in triplicate. Thrombin generation was monitored also following supplementing PFP with adiponectin or leptin (BioVision, San Francisco, USA), with fibrinogen (Sigma-Aldrich, St Louis, USA), or with palmitic acid or linoleic acid (Sigma-Aldrich).

### Coagulation and circulating parameters

Prothrombin and FVIII were measured in PFP samples diluted 1:40–80 in factor diluent (Instrumentation Laboratory, Le Pré Saint Gervais, France). For each assay 50 μl of diluted sample were added to 50 μl of human prothrombin-deficient plasma (Siemens Healthcare Diagnostics SAS, Saint-Denis, France) or FVIII deficient plasma (Dade Behring, Deerfield, USA). After 1 min of incubation at 37°C in a KC10 coagulometer, coagulation was started by addition of 80 μl of Thromborel® S. Calibration curves were generated using the reference plasma Unicalibrator (Diagnostica Stago, Asnières, France). Fibrinogen was measured in PFP samples diluted 1:10–20 in Owren–Koller buffer (Diagnostica Stago, Asnières, France). Unicalibrator was used to generate calibration curves. After 4 min of incubation at 37°C in a KC10 coagulometer, coagulation was started by addition of 100 μl of Fibriquik (Biomérieux-Trinity Biotech, Bray, Ireland). Antithrombin levels were measured with the Coamatic® antithrombin test kit from Chromogenix, and TAT with the Enzygnost® TAT micro (Instumentation Laboratory). TF and TF pathway inhibitor (TFPI) activities were measured in PFP using the Actichrome® tissue factor and Actichrome® TFPI activity assay respectively (American Diagnostica, Stamford, CT). PAI-1 levels were measured with the rat PAI-1 total antigen ELISA kit from Innovative Research, Inc. IL-13 and IL-1β concentrations were measured with the IL-13 and IL-1 beta rat ELISA kits from Invitrogen. MMP-9 levels were measured with the Quantikine rat total MMP-9 immunoassay from R&D Systems. VCAM-1 was assessed with the rat VCAM-1 ELISA kit from Elabscience.

### *In vitro* fibrinolytic test

PFP (20 μl) was diluted by addition of 40 μl buffer containing 5 pM recombinant TF, PV at 4 μM equivalent PS, 5 nM rabbit thrombomodulin (TM) (American Diagnostica, Greenwich, USA) and 4 μg/ml recombinant human tissue Plasminogen Activator (tPA) Actilyse® (Boehringer Ingelheim, Ingelheim am Rhein, Germany). Clot formation was initiated by addition of 10 μl of 100 mM CaCl_2_. To monitor clot lysis, absorbance was read kinetically at 405 nm using a microplate reader. To standardize the figure, for each sample basal optical density (OD) after lysis was subtracted from each point of the curve. Half lysis time was defined as the time required to reach half-maximal variation in OD.

### Microscopy of fibrin fiber ultrastructure

The thrombin generation assay was performed in order to generate fibrin for fixation using the same TF and PV concentrations as in the CAT experiments. This was done using plasma on paper disks and a Rhodamine substrate was used (Ninivaggi et al., 2012). Immediately after thrombin generation was finished (50 min for each run), the mineral oil was removed from the well and a solution of glutaraldehyde (grade I) in phosphate buffered saline (PBS) (Sorensen's PBS, pH 7.2) was applied. This was put at room temperature for 1 h and then kept at 4°C overnight. The samples were then washed 5 times with PBS and a secondary fixation was performed in OsO_4_ (1%) in sodium cacodylate (200 nM, pH 7.4) for 1 h at RT. The samples were then dehydrated with increasing concentrations of ethanol each during 3 min (30, 50, 70, 90, 100%) and the last step (100%) was performed three times. Further dehydration was accomplished by a hexamethyldisilazane (HMDS)/ethanol solution (1:1) for 3 min and HMDS for 10 min. The samples were removed from the wells and left to dry. In order to visualize the samples with a Phenom G2Pro scanning electron microscopy (SEM) (Phenom-World, Eindhoven, the Netherlands), they were put on stubs using carbon tabs and coated with gold.

For each sample, 3–5 pictures were analyzed. Fiber thickness was measured using ImageJ software (version 1.48v). For each picture 100 measurements were performed. The density of the fibers was calculated from the pictures by counting the number of fibers that crossed a line of 26.8 μm (Konings et al., 2011).

### Rat cytokine antibody array

The Rat Cytokine Array Panel A (Cat # ARY008) from R&D system (Minneapolis, MN) was used to probe cytokines in PFP from MSZR and LZR by following the procedures recommended by the manufacturer. Bound antibodies were detected by chemiluminescence using the Immobilon™ Western Chemiluminescent HRP Substrate (Millipore, Billerica, MA). This was performed once with a plasma pool from 5 to 6 animals to reduce inter-animal variability in each group.

### Phospholipid procoagulant activity

The chromogenic assay measuring the phospholipid-related procoagulant activity (PPA) in VSMCs was performed as described previously for plasma (Wagenvoord et al., 1994; Membre et al., 2008). VSMCs cultured in 96 well plates were washed and 50 μl of 50 mM Tris, 175 mM NaCl, pH 7.9 (TBS) containing 2 g/l bovine serum albumin (BSA) were added as well as 50 μl of activated factor X (1.2 nM), activated factor V (2.4 nM), CaCl_2_ (15 mM) and 50 μl of bovine prothrombin (6 μM) plus Z-Gly-Gly-Arg-AMC substrate (1.25 mM) in 20 mM HEPES pH 7.5 containing 60 g/l BSA. The plate was placed in the Fluoroskan Ascent fluorometer and allowed to warm up to 37°C for 5 min before kinetic readings were taken over 10 min. Phospholipid concentration was estimated from the initial rate of thrombin formation by reference to a standard curve constructed with PV, and expressed as PS equivalents.

### Western blot

Cell extracts were obtained by lysing VSMCs in complete Lysis-M buffer (Roche Diagnostics Corporation, Basel, Switzerland). Detergent-soluble fractions were retained, and protein concentrations in samples were determined using a Bradford protein assay (Bio-Rad, Hercule, USA). Lysates containing 30 μg of protein were electrophoresed on polyacrylamide gels (8% gel), transferred to Hybond-C nitrocellulose membranes (transblot turbo, Bio-Rad, Hercule, USA) and blotted with the following antibodies: α-smooth muscle actin (αSMA), 4/1,000 (Sigma-Aldrich), smooth muscle myosin heavy chain (SM-MHC), 1/1,000 (Abcam; Cambridge, UK); smoothelin, 1/500 (Santa Cruz Biotechnology, USA); integrin α_v_, 1/1,000 (Santa Cruz Biotechnology, Dallas Texas); integrin β_3_ 1/500 (Merck Millipore, Billerica, USA) and tubulin, 2/1,000 (Sigma-Aldrich). After rinsing, incubation with a secondary rabbit antibody 1/1,000 (α_v_, β_3_, smoothelin, SM-MHC, Sigma-Aldrich) and mouse antibody 1/1000 (αSMA, tubulin, Sigma-Aldrich). Reactions were visualized by the ECL Western Blot Detection Kit (Bio-Rad, Hercule, USA) after incubation with peroxidase conjugates 1/2000 (GE Healthcare, Little Chalfont, UK). Tubulin was used as loading control and the protein expression was normalized to tubulin.

### *In situ* gelatin zymography

*In situ* gelatin zymography was performed to determine the gelatinase activity across the aortic wall using DQ-gelatin (Life Technologies, Paisley, UK) as described previously (Mook et al., 2003). Fluorescein isothiocyanate (FITC, 1/110), and 4′,6-diamidino-2-phenylindole (DAPI, 1/150) filters were used to visualize the degree of gelatinase activity and the localisation of nuclear tissue by fluorescence microscopy using a x20 optical objective (Keynece, Osaka, Japan). Analysis of average fluorescence was performed for three 20 μm thick profile lines across 3 arterial wall regions for each sample.

### Zymography analysis

VSMCs from LZR or MSZR (passage 4–6) were seeded (50,000 cells/well) in 6-well culture plates in DMEM/F-12 supplemented with 10% fetal bovine serum (life technology Thermo Fisher Scientific, Waltham, USA). Cells were grown to subconfluence and after 16 h in serum-free medium, cells were washed with PBS (Sigma-Aldrich), the medium was changed and cells were incubated for 4, 8, or 20 h at 37°C. Conditioned media were then removed and centrifuged at 500 g for 10 min at room temperature and used for the determination of MMP-2 secretion.

Conditioned media were analyzed for gelatin degradation by electrophoresis under non-reducing conditions on a 10% polyacrylamide-SDS gel containing 0.1% gelatin. Gels were washed for 1 h at room temperature in a 2% triton X-100 solution and incubated overnight at 37°C in 50 mM Tris–HCl/10 mM CaCl_2_ (pH 7.6) buffer.

Gels were stained in a 0.1% coomassie Blue (G250)/45% methanol/10% acetic acid solution and de-stained in a 10% acetic acid/20% methanol solution. White lysis strips, indicative of gelatinolytic activity, were revealed and scanned (Fujifilm LAS 4000, Life sciences, Branford, USA). Densitometric analysis was made using MultiGauge software (Fuji, Tokyo, Japan). Fetal bovine serum diluted at 1% in serum free medium was used as a positive control.

### Statistical analysis

Results are presented as mean ± standard error of the mean. Data were analyzed by a one-way or two-way ANOVA, followed by a Fisher's test for multiple comparisons to evaluate the influence of age and strain and their interaction on the different variables. In the case of SEM measurements, the differences in fiber thickness were analyzed using the Mann Whitney *U*-test.

## Results

### Platelet aggregation, thrombin generation, and fibrinolysis were all impaired with the MetS and/or aging

Platelet count in blood was increased in MSZR at both ages compared to the same aged LZR (Table 1). Platelet aggregation using washed platelets and collagen as a strong agonist was not significantly modified as shown by the mean maximum aggregation (Figure 1A). For platelet aggregation in PRP using ADP, mean maximum aggregation was increased in 80 week-old MSZR and LZR compared to 25 week-old controls (Figure 1B). The F1+2 fragment was analyzed to evaluate the *in vivo* reactivity of the coagulation system. The amount of F1+2 fragment was increased in 25 week-old MSZR compared to the same aged LZR (Table 1). Thrombin generation measurement was performed as an integrative *in vitro* phenotype of coagulation. Adult and very old MSZR had a significantly increased endogenous thrombin potential (ETP) compared to same aged LZR. The other thrombin generation parameters (lag time, peak, and velocity) were not changed significantly except for the time to peak which was increased in obese at both ages (Table 1; Figure 1C). The ratio of thrombin generation in PFP and PRP compared to 25 week-old LZR was made to evaluate the platelet reactivity impact on thrombin generation. Interestingly, thrombin generation was more increased in PRP from MSZR at 25 week of age compared to 80 week-old rats (Figure 1D). The coagulation parameters, TF, TFPI, prothrombin and fibrinogen, were all increased in MSZR compared to LZR at both ages. TFPI was decreased and fibrinogen was increased with age in MSZR and prothrombin was increased with age in LZR. FVIII was increased significantly with age and MetS in 80 week-old MSZR. Antithrombin measurements showed no modification in MSZR and LZR rats (Table 1). Fibrin clots were characterized by SEM. Computerised analysis of the SEM images showed a decrease of fibrin fiber thickness in MSZR compared to LZR at both ages while fiber density was only increased in 80 week-old LZR (Figures 1E-G). Circulating levels of PAI-1 were increased in both 80 week-old LZR and MSZR (Figure 1H). In a fibrinolysis test (Figure 1I), half-time lysis was increased in MSZR compared to LZR at both ages and aging significantly increased half-time lysis in both groups (Figure 1J). Maximal lysis speed was not modified (Figure 1K).

**Table 1:** Blood coagulation parameters and thrombin generation parameters of LZR and MSZR at 25 and 80 weeks of age.

	**25 week-old**		**80 week-old**		**ANOVA**		
	**LZR**	**MSZR**	**LZR**	**MSZR**	**Strain**	**Age**	**Interaction**
*n*	9	10	12	9			
Platelets (10^3^/mm^3^)	574 ± 37	789 ± 34^*^	633 ± 29	834 ± 63^*^	≤0.0001	0.009	0.013
F1+2 (pmol/l)	4.1 ± 0.5	7.9 ± 1.0^*^	5.8 ± 1.2	5.5 ± 0.9	0.009	0.7	0.05
TF (pM)	0.3 ± 0.1	12.2 ± 1.7^*^	2.0 ± 0.4	9.9 ± 1.5^*^	≤0.0001	0.8	0.09
TFPI activity (U/ml)	4.9 ± 0.2	11.2 ± 0.2^*^	5.4 ± 0.2	9.9 ± 0.6^*^^#^	≤0.0001	0.3	0.01
FVIII (%)	104 28	190 34	124 28	466 52^*^^#^	≤0.0001	0.002	0.001
Prothrombin (%)	94 ± 3	223 ± 19^*^	155 ± 14^#^	264 ± 16^*^	≤0.0001	0.002	0.5
AT (%)	129 ± 2	125 ± 2	127 ± 1	123 ± 3	0.04	0.5	0.9
Fibrinogen (g/l)	2.8 ± 0.1	4.0 ± 0.2^*^	3.1 ± 0.1	4.9 ± 0.2^*^^#^	≤0.0001	0.0003	0.2
*n*	11	11	10	7			
Lag time (min)	1.5 ± 0.1	1.7 ± 0.1	1.4 ± 0.1	1.5 ± 0.1	0.09	0.4	0.6
Peak (nM)	99 ± 8	121 ± 9	102 ± 10	117 ± 15	0.07	0.98	0.7
Time to peak (min)	4.4 ± 0.1	5.2 ± 0.3^*^	4.1 ± 0.1	5.3 ± 0.2^*^	≤0.0001	0.6	0.3
ETP (nM.min)	395 ± 37	549 ± 52^*^	362 ± 34	553 ± 76^*^	0.001	0.8	0.8
Velocity (nM/min)	35 ± 3	37 ± 4	40 ± 4	31 ± 4	0.6	0.98	0.2

*Results are mean ± standard error to the mean*.

* p < 0.05, SMZR vs. LZR at the same age;

# *p < 0.05, 80 vs. 25 week-old rats in the same strain. F1+2, fragment 1+2; TF, tissue factor; TFPI, tissue factor pathway inhibitor; AT, antithrombin; ETP, endogenous thrombin potential*.

**Figure 1:**
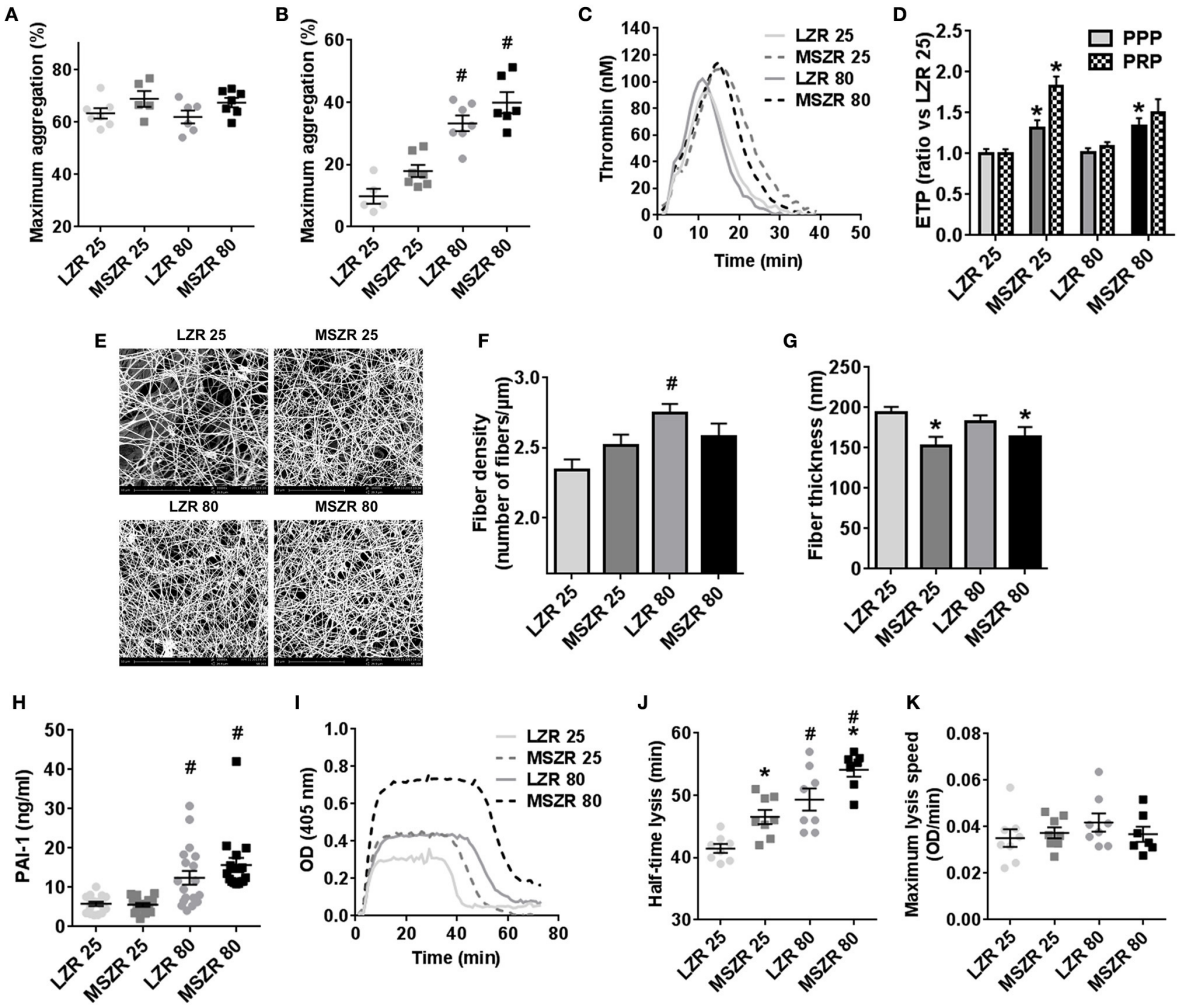
Platelet aggregation, thrombin generation, and fibrinolysis in LZR and MSZR rats**. (A)** Mean maximum aggregation in washed platelets in response to collagen (5 μg/ml) and in **(B)** platelet-rich plasma (PRP) in response to ADP (5 μM), with the platelet count adjusted to 200 × 10^9^ platelets/l. **(C)** Calibrated automated thrombinography (CAT) in rat plasma. Mean thrombin generation curves in platelet free plasma (PFP) triggered by 5 pM tissue factor in LZR and MSZR at 25 and 80 weeks of age. **(D)** Endogenous thrombin potential (ETP) in PFP and PRP of 25 and 80 week-old LZR and MSZR, expressed as ratios of values for 25 week-old LZR. **(E)** Ultrastructure of fibrin fibers was visualized by scanning electron microscopy. Pictures were made at 10,000 × magnification. **(F,G)** Fiber thickness and fiber density of fibrin clot in LZR and MSZR. **(H)** ELISA results of PAI-1 measured in PFP (*n* = 17–19). **(I)** Representative curves of fibrinolytic tests in PFP in LZR and MSZR. **(J,K)** Half-lysis time and maximal lysis speed of fibrinolytic tests in LZR and MSZR. Results are mean ± standard error of the mean (*n* = 7–11). ^*^p < 0.05 vs. LZR at the same age; ^#^*p* < 0.05 vs. 25 week-old rats in the same strain.

### Inflammation, metabolic factors, and free fatty acids modified thrombin generation

**Figure 2:**
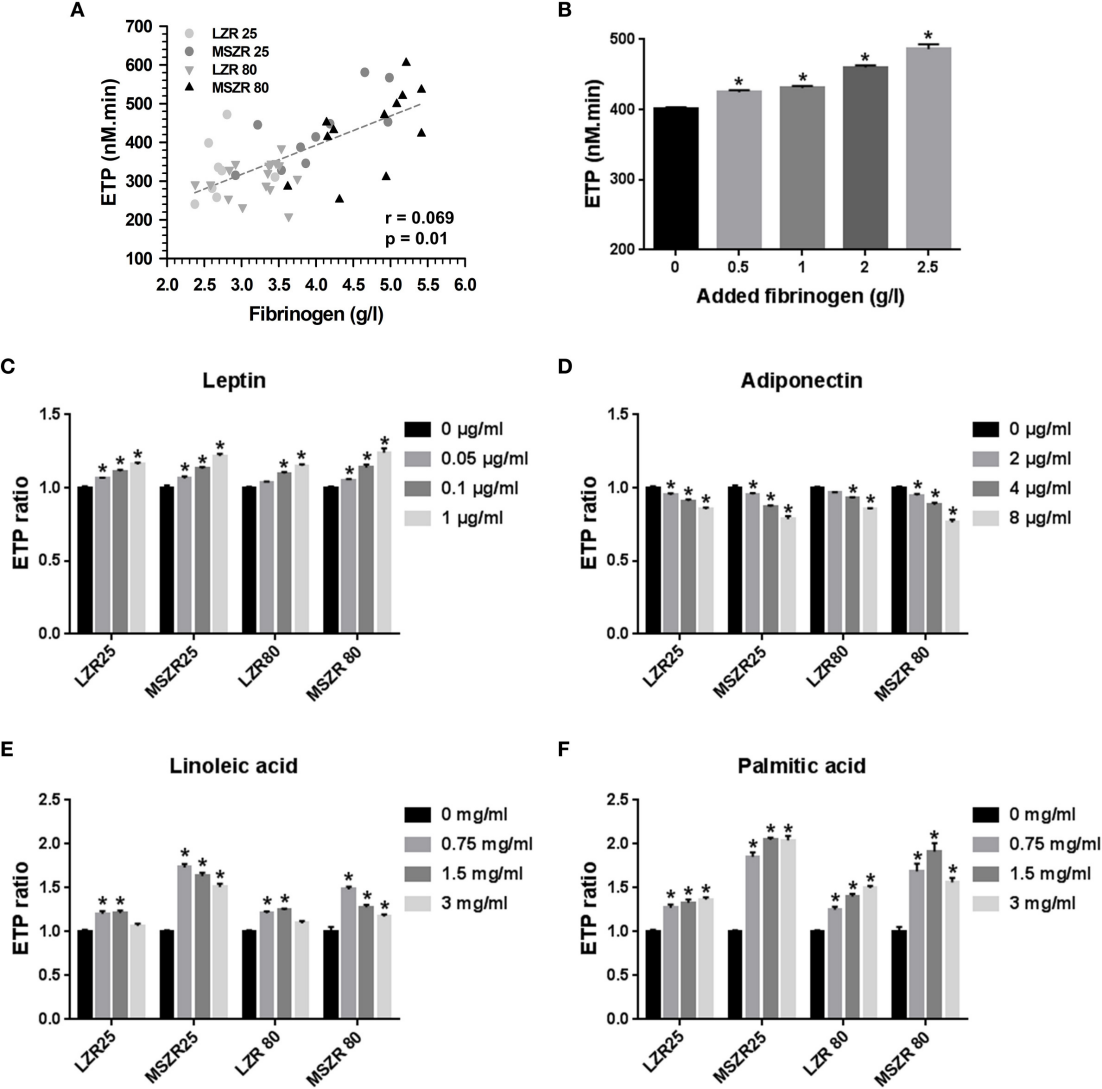
Effect of fibrinogen, adipokines, and free fatty acids on thrombin generation. **(A)** Correlation between ETP and plasma fibrinogen concentration of 25 and 80 week-old LZR and MSZR (*r* = 0.069, *p* = 0.01). **(B)** ETP values in 25 week-old LZR platelet free plasma supplemented with 0.5, 1.0, 2.0, or 2.5 g/l fibrinogen. **(C–F)** ETP values, expressed as ratios of values in presence of adipokines or free fatty acids to those obtained with no addition for each group, in platelet free plasma supplemented with 0.05, 0.1, or 1.0 ng/ml leptin **(C)**, with 2, 4, or 8 μg/ml adiponectin **(D)**, with 0.75, 1.5, or 3 mg/ml of linoleic acid **(E)** or with 0.75 1.5, or 3 mg/ml of palmitic acid **(F)**. Results are mean ± standard error of the mean (*n* = 11–16). ^*^p < 0.05 vs. no addition.

Fibrinogen concentration was correlated highly to ETP (*r* = 0.069) and supplementing plasma with exogenous fibrinogen at concentrations that agreed with the changes between MSZR and LZR gradually increased ETP (Figures 2A,B). The 1.2-fold increase in ETP with the 2.5 mg/mL concentration is consistent with the 1.4 increase in plasma fibrinogen in MSZR. We have then tested the effects of addition of exogenous leptin, adiponectin, linoleic acid, and palmitic acid to PFP at concentrations selected to encompass the range previously reported for each molecule in MSZR (Sloboda et al., 2012; Godin et al., 2013). Addition of leptin or adiponectin elicited similar concentration-dependent changes in ETP whatever the group of rat. The two adipokines had opposite effects on thrombin generation, leptin increased ETP whereas adiponectin decreased it (Figures 2C,D). The two lower concentrations of added linoleic acid (0.75 and 1.5 mg/mL) had clear procoagulant effects whereas the higher concentration (3 mg/mL) was less effective in increasing thrombin generation (Figures 2E,F). There was a significant increase in thrombin generation for all added concentrations of palmitic acid whatever the group of rat. The results show an additive effect of FFAs on MSZR plasma.

### Plasma cytokines were increased both with MetS and aging

**Figure 3:**
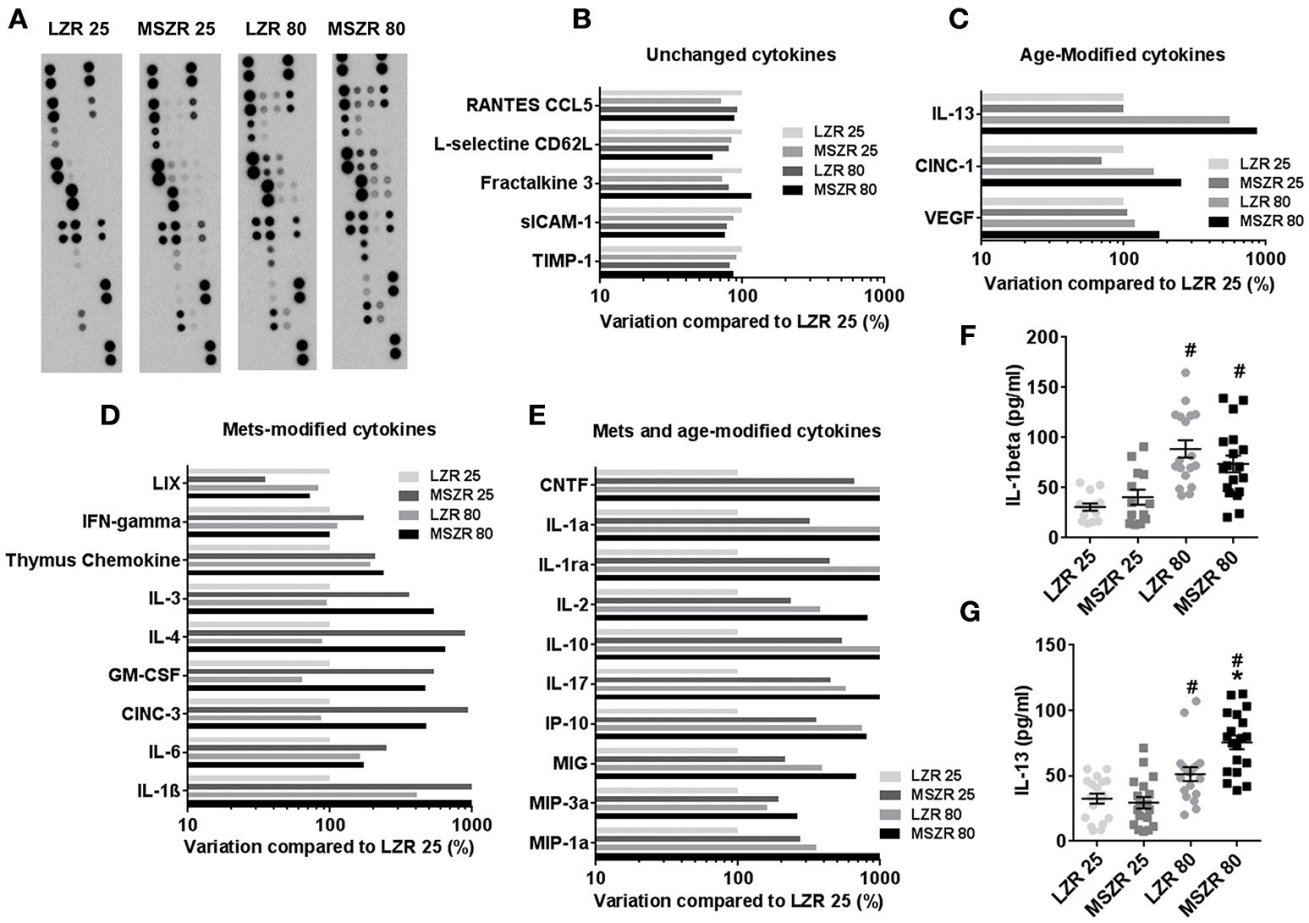
Plasma cytokine array in Zucker rats**. (A)** Cytokine arrays of pooled platelet free plasma from 25 to 80 week-old MSZR and LZR. Relative chemoluminescence compared to 25 week-old LZR was measured. **(B)** Unchanged cytokines, **(C)** cytokines modified with age, **(D)** with MetS, or **(E)** both with MetS and age. ELISAs results for IL-1β **(F)** and IL-13 **(G)** measured in PFP (*n* = 14–18), results are mean ± standard error of the mean, ^*^p < 0.05 vs. LZR at the same age; ^#^*p* < 0.05 vs. 25 week-old rats in the same strain. VEGF, vascular endothelial growth factor; CINC-1, cytokine-induced neutrophil chemoattractant 1; CINC-3, cytokine-induced neutrophil chemoattractant 3; GM-CSF, granulocyte macrophage colony stimulating factor; MIP, Macrophage Inflammatory Protein; MIG, C-X-C motif ligand 9; IP-10, interferon gamma-induced protein 10; CNTF, ciliary neurotrophic factor; INFγ, interferon γ; IL, interleukin.

To explore inflammation in our model we performed a plasma cytokine array of 27 cytokines in order to provide qualitative data that will subsequently be used to quantify cytokines known likely to promote prothrombotic phenotypes (Figure 3). Panel A presents pictures of the cytokine array membranes. A 50% variation between two groups was chosen as a threshold to classify cytokines into four groups. The first group of five cytokines showed no modifications (Figure 3B), a second group of eight cytokines were increased with MetS (Figure 3C), a third group of three cytokines were increased with aging (Figure 3D) and a last group of 11 cytokines were increased with both MetS and aging (Figure 3E). The highest variation between 25 week-old MSZR and LZR was found for IL-1β (>3,000% variation) and the highest variation between 80 and 25 week-old rats was observed for IL-13 (>400% variation). ELISAs performed with individual rat PFP for IL-1β and IL-13 showed an increase of these cytokine levels in LZR and MSZR with age (Figures 3F,G). IL-13 was increased also in 80 week-old MSZR compared to same aged LZR.

### MetS and aging-induced inflammation and haemostasis impairment were related to alteration of VSMCs

**Figure 4:**
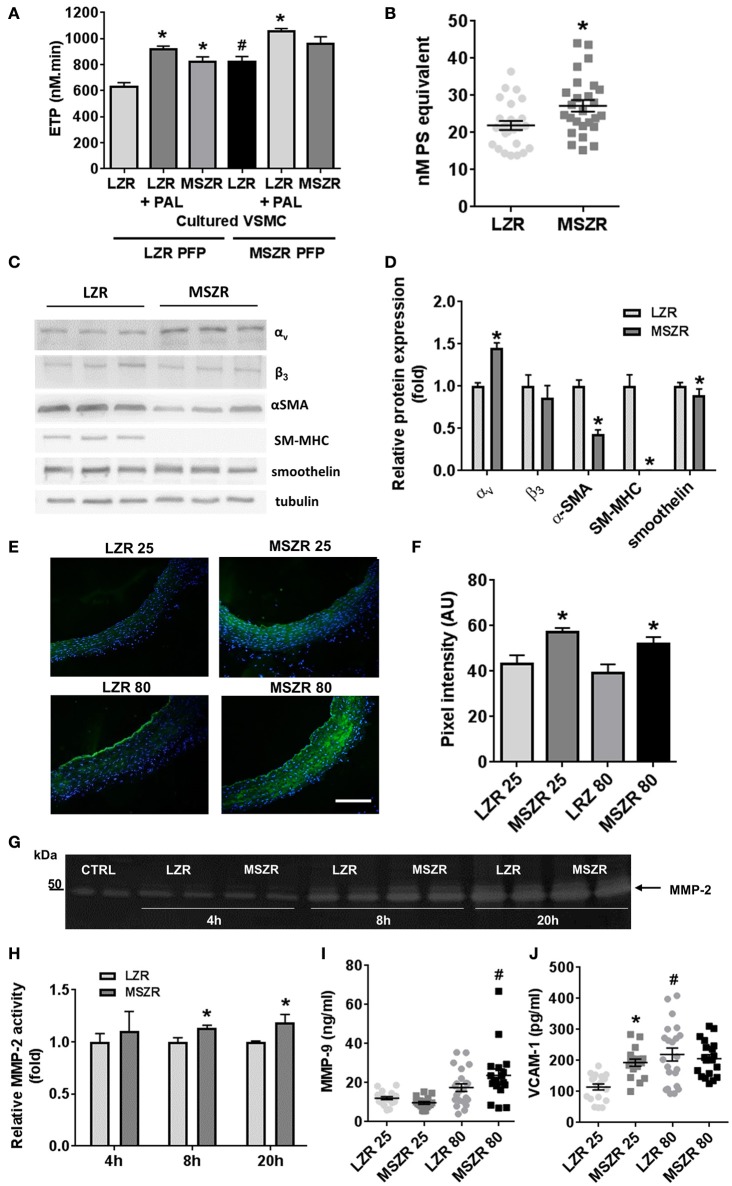
Role of smooth muscle cells in thrombin generation. **(A)** ETP values measured at the surface of vascular smooth muscle cells (VSMCs) from LZR and MSZR, with LZR or MSZR platelet free plasma (PFP), and with or without 1.5 g/l exogenous added palmitic acid (PAL). Results are mean ± standard error of the mean, *n* = 3 with 6 wells per condition per experiment. ^*^p < 0.05 vs. LZR VSMC, ^#^*p* < 0.05 vs. LRZ PFP and LRZ VMSC. **(B)** VSMC associated procoagulant activity reported as phosphatidylserine (PS) equivalent in LRZ and MSZR. Results are mean ± standard error of the mean (*n* = 25). ^*^p < 0.05 vs. LZR. **(C)** Typical Western blot and **(D)** quantification analysis of VSMC differentiation markers (αSMA, SM-MHC, and smoothelin) and integrin subunits (α_v_ and β_3_) in cultured VSMCs. Results, expressed as fold change vs. VSMCs from LZR, are mean ± standard error of the mean (*n* = 6). ^*^p < 0.05, MSZR vs. LZR. **(E)** Representative images of gelatinolytic metalloproteinase activity in the aorta was measured using *in situ* gelatin zymography for each group of Zucker rats. Fluorescence as marker for intra-plaque gelatinolytic activity was quantified. Nuclei were visualized by DAPI staining. **(F)** Average wall fluorescence of the gelatinolytic metalloproteinase activity in the aorta. **(G)** Representative images of zymography gels of LZR and MSZR VSCMCs supernatant at 4, 8, and 20 h. **(H)** Relative MMP-2 activity in LZR and MSZR VSMC supernatant at 4, 8, and 20 h. Results are mean ± standard error of the mean (*n* = 5). ^*^p < 0.05, MSZR vs. LZR. ELISAs results of MMP-9 **(I)** and VCAM-1 **(J)** measured in PFP (*n* = 17–22). ^*^p < 0.05 vs. LZR at the same age; ^#^*p* < 0.05 vs. 25 week-old rats in the same strain.

To explore the contribution of VSMCs, thrombin generation was measured at the surface of cultured VSMCs isolated from LZR and MSZR. Thrombin generation with PFP from LZR and MSZR was always increased at the surface of MSZR VSMCs compared to LZR VSMCs. Remarkably, addition of palmitic acid in LZR VSMCs increased thrombin generation to the level of MSZR independently of the PFP used (Figure 4A). MSZR VSMCs displayed increased procoagulant phospholipids at their surface compared to LZR VSMCs (Figure 4B). Integrin subunit α_v_ was increased in MSZR compared to LSZ VSMCs while the β_3_ subunit was not modified. VSMC differentiation markers α-SMA, SM-MHC, and smoothelin, interestingly, were all decreased in MSRZ VSMCs compared to LZR VSMCs (Figures 4C,D). Thus, *in situ* gelatin zymography was performed to explore MMP activity through gelatinase activity (Figure 4E). Figure 4E shows representative photographs of *in situ* gelatin zymography in aorta, gelatinase activity is in green. Mean gelatinase activity in the aortic wall was increased in 25 and 80 week-old MSZR compared to age matched LZR aortas (Figure 4F). However, age did not modulate gelatinase activity. At the cellular level MSZR VSMCs displayed increased MMP-2 secretion compared to LZR VSMCs (Figures 4G,H). Circulating levels of MMP-9 were increased in 80 week-old MSZR whereas VCAM-1 was increased in 25 week-old MSZR compared to same aged LZR and in 80 week-old LZR (Figures 4I,J).

## Discussion

The aim of the present study was to determine concomitant changes in the haemostasis system and VSMC phenotype and their interplay with FFAs and MMPs during aging in obese rats compared to lean rats of the same age. Our results demonstrated (1) increased thrombin generation in MetS in plasma as early as 25 weeks of age, independently of platelets and at the surface of VSMCs; (2) reinforcement of this hypercoagulability by reduced plasma fibrinolysis; (3) no influence of aging on plasma thrombin generation; (4) an age-related increase in platelet aggregation and clot half lysis time and, (5) contribution of saturated FFAs to the increased thrombin generation both in plasma and at the surface of VSMCs.

Increased thrombotic risk can be attributed to three factors: abnormalities in the vessel wall, in blood flow, and in haemostasis including coagulation and fibrinolysis. We found previously that MSZR presented endothelial dysfunction as shown by increased circulating VWF. This endothelial dysfunction was exacerbated during aging as shown by increases in both VWF and soluble CD146 (Sloboda et al., 2012).

Few studies have used Zucker rats to look at haemostasis and to our knowledge none have been performed in very old Zucker rats. Paul et al. found that 12 week-old diabetic Zucker rats presented unmodified *in vitro* platelet reactivity (Paul et al., 2007). Recently Shang et al. have shown increased thrombosis, increased thrombin generation and decreased fibrinolysis in 7–10 week-old diabetic Zucker rats (Shang et al., 2014). They found also decreased platelet reactivity to collagen and ADP in obese rats in PRP. In PRP, we found increased platelet aggregation using ADP in 80 week-old MSZR and LZR rats compared to 25 week-old controls, but not between rats of the same age. In addition, we were not able to aggregate platelets using collagen. Washed platelets were able to aggregate when triggered with collagen but we did not find any significant changes with obesity or with age. These changes might be related to the metabolic differences existing between rats since they used diabetic Zucker rats while we used obese Zucker rats that only develop diabetes very late with age. Moreover, platelet count was not modified in the diabetic Zucker rats of the Shang et al. study while we found a 25% increased count in MSZR compared to LZR at both ages. Interestingly, platelet-related thrombin generation showed a very important increase in 25 week-old MSZR compared to thrombin generation made with PFP. Altogether, increased platelet aggregation to ADP with age concomitant to increased platelet count in obese Zucker rats is in favor of a prothrombotic state.

To better assess the prothrombotic state in obese and aged rats we investigated *in vivo* thrombin generation by measuring F1+2 fragments, which were increased in MSZR indicating increased *in vivo* formation of thrombin with MetS. As expected, MetS also increased the *in vitro* thrombin generation capacity of plasma, but this ability was not modified with age. This change in the *in vitro* reactivity of the coagulation system points out the role of several components including metabolic factors and the vascular wall. Regarding individual clotting factors it was clear that TF increased in MSZR as well as its inhibitor (TFPI). Increased prothrombin concentration leads to higher thrombin generation and can contribute to the increased ETP in MSZR. Other procoagulant factors such as FVII, FVIII, and VWF are known to be increased with MetS and aging. Metabolic factors such as leptin and adiponectin can participate in haemostasis. Leptin has been suggested previously to represent a link between obesity and atherothrombosis (Petrini et al., 2016). It has been reported that leptin enhanced platelet aggregation while adiponectin reduced it (Konstantinides et al., 2001; Restituto et al., 2010). Adiponectin has been involved also in the endothelium anticoagulation function (Lee et al., 2011) since it increased endothelial TFPI synthesis (Chen et al., 2008). We found in all Zucker rats a strong positive correlation between plasma TPFI and adiponectin concentrations dosed previously (data not shown; Sloboda et al., 2012). Moreover, in our study, we found for the first time that leptin increased ETP and that adiponectin decreased it. Despite it being a modest effect, it argues for a major involvement of adipokines in the regulation of thrombin generation.

Fibrinogen concentration was correlated also to ETP and we confirmed that increased plasma fibrinogen increased ETP (Kumar et al., 1994). Thrombin linked to fibrin can possibly be protected from inhibition by antithrombin, in the same way as it is protected from inhibition when bound to TM (Bourin, 1987). This may participate in explaining the increased time to peak observed in MSZR and increased ETP with no significantly increased peak.

We found that fibrinogen concentration was increased in MSZR and during aging. In favor of the relevance of this result it has been shown that synthesis of fibrinogen is upregulated by inflammatory cytokines such as IL-6 (Morozumi et al., 2009). The consequence of an increased thrombin generation was an increased fibrin network formation in MSZR as shown by thinner fibrin fibers (Wolberg, 2007). The increase in PAI-1 with aging in LZR as well as in MSZR is relevant to human physiology since it is known that during aging PAI-1 is associated with an increased thrombotic risk. In addition, the fibrinogen concentration increased during aging but the mechanisms underlying this association with thrombotic risk are unclear (Cesari et al., 2010). Human fibrinolysis is also impaired in the MetS with a decrease in clot lysis ability linked to increased PAI-1 (Pandolfi et al., 2001). Organization of the fibrin network is likely due to the increased thrombin generation found in MSZR (Wolberg, 2007). Moreover, clots with thinner fibrin fibers are more resistant to lysis than clots with thick fibers (Gabriel et al., 1992). This is supported by the increased half-time lysis found in MSZR and very old Zucker rats. Other factors must be implicated since fiber thickness was unchanged with age in both groups whereas fibrinolysis time increased only during aging indicating the formation of a denser clot. In line with this, adiponectin may act as an anticoagulant molecule. Indeed, full length adiponectin reduces platelet aggregation, inhibits TF and enhances TFPI expression at the surface of endothelial cells (Chen et al., 2008; Restituto et al., 2010). Both adiponectin and IL-13 increase the expression of MMPs which can degrade fibrinogen (Hotary et al., 2002; Wanninger et al., 2011; Firszt et al., 2014). Consistent with this, we found an increase in IL-13 plasmatic concentration with aging and also with the MetS in 80 week-old MSZR which presents the same variations as plasma levels of MMP-9 and FVIII. Whether adiponectin interplays directly with fibrinogen remains an open question. The increase in FVIII with MetS and associated inflammatory stimuli was anticipated in Zucker rats as it is in humans (Begbie et al., 2000; Kotronen et al., 2011).

Inflammation during aging and in the MetS triggers vascular remodeling. Fibrinogen (Lominadze et al., 2010) as well as fibrin and fibrin degradation products have proinflammatory functions that can modify VSMC phenotype (Lu et al., 2011). Cytokines in the plasma, as shown in the array presented here, are increased by the MetS, aging, or both. Our data indicated that the more relevant proinflammatory cytokines such as IL-1α, IL-1β, IL-2, IL-3, and IL-6 were increased early with the MetS while few anti-inflammatory cytokines were increased with MetS and aging (IL-10, IL-1ra, IL-17). Our cytokine array made with a pool of plasma for each group was checked using ELISA measurements with individual samples for the two main cytokines involved in the regulation of haemostasis (IL-13 and IL-1β). IL-13 changes were confirmed while IL-1β increased only with aging but not with the MetS at 25 weeks of age. This points to a determinant role of age in complex vascular pathologies including several comorbidities. IL-1β has a pleiotropic effect in the development of atherothrombosis through its action on leukocyte adhesion to the vascular wall and induction of procoagulant activity (Libby et al., 1986; Dinarello, 2011). Recently, inhibition of IL-1β and subsequent reduction of inflammation (without modification of lipid levels) in patients with previous episodes of myocardial infarction was found to reduce recurrent cardiovascular events (Ridker et al., 2017). These findings are in line with the increase of circulating IL-1β and increased activity of haemostasis with age we observed in MSZR. Therefore, exploration of haemostasis function in MSZR with inhibition of IL-1β could be of interest.

Other factors related to MetS that can potentiate the modifications we observed in MSZR haemostasis are FFAs. Saturated FFAs such as palmitic acid are known to be associated with ischemic heart disease and increase postprandial concentrations of fibrinogen (Simon et al., 1995; Pacheco et al., 2006). One other mechanism proposed recently to explain the thrombogenic effect of palmitic acid was its ability to induce extracellular release of histones (Shrestha et al., 2013). Histones are known to promote thrombin generation through platelet activation (Semeraro et al., 2011). Additionally, palmitic acid was measured recently in diabetic Zucker rats pointing out a 2.75 times increased concentration in obese rats (0.68 g/l in LZR vs. 1.87 g/l in MSZR) (Godin et al., 2013). A similar increase was observed for a polyunsaturated FFA, linoleic acid. We supplemented 25 week-old LZR PFP with linoleic or palmitic acid to reach MSZR plasma concentrations. We showed for the first time a direct effect of FFAs on thrombin generation confirming the prothrombotic effect of palmitic acid.

All these FFAs, pro-inflammatory cytokines and coagulation factors can have deleterious effects on the vascular wall. We have shown previously the presence of endothelial dysfunction in MSZR (Sloboda et al., 2012). In the present study we studied VSMCs in more detail. Interestingly, thrombin generation measured at the surface of VSMCs from MSZR was increased compared to LZR VSMCs. This increase can be related to the increased procoagulant phospholipids at the surface of MSZR VSMCs. We showed recently that thrombin generation at the surface of VSMC from spontaneously hypertensive rats (SHR) leads to increased ETP and VSMCs were responsible for a prothrombotic phenotype in SHR rats. In the same way as for SHR rats, increased VSMC-supported thrombin generation can be a mechanism implicated in the prothrombotic phenotype we have observed in Zucker rats. In these cellular experiments addition of palmitic acid exacerbated also thrombin generation over MSZR VSMCs.

MMPs are related to FFAs, obesity-related diseases such as type 2 diabetes and overall, inflammation. In our model, mean gelatinase activity, focusing on MMP-2 and-9 activities, was increased in MSZR. These molecules are responsible for the degradation of type IV collagen, elastin, fibronectin, and laminin, among other proteins. It is known that FFAs and insulin lead to hyperactivity of MMP-2 and-9 (Boden et al., 2008). The close relation between MMPs and insulin was demonstrated also in Zucker rats (Zhou et al., 2005). IL-13 was increased in old rats and is known to be an activator of MMPs (Firszt et al., 2014). This increase in aortic MMP activity in the intima with aging has been described in rats and was 2-fold higher in old vs. young non-human primates (Li et al., 1999; Wang et al., 2007). In addition, MMP activity may participate also in age-related vascular remodeling in the aortic media since MMPs accumulate around elastic fibers in the aortic media (Li et al., 1999), which become fragmented with age-associated increases in arterial stiffness which thus increases cardiovascular risk. Interestingly, MMP production can be stimulated through integrin α_v_β_3_ (Bendeck et al., 2000). Concerning this pathway, we found an increase of the α_v_ subunit in MSZR VSMCs and MMP-2 secretion was increased in MSZR compared to LZR. Moreover, we have shown previously that this integrin is responsible for thrombin generation supported by VSMCs and it argues for its role in vascular remodeling (Mao et al., 2012). Very interestingly all VSMC differentiation markers we tested were downregulated in MSZR and even absent concerning SM-MHC. This illustrates a phenotype switch from contractile to secreting VSMCs occurring in vascular diseases such as atherosclerosis (Lacolley et al., 2012).

In conclusion, our study demonstrates in MetS a prothrombotic phenotype of the blood compartment reinforced by procoagulant properties of the vascular wall. Regarding the mechanisms, fibrinogen contributes to this hypercoagulable phenotype in plasma at an early stage of MetS. Leptin and adiponectin exert moderate opposite effects on thrombin generation precluding a major contribution of adipokines. An increase in proinflammatory cytokines likely increased MMP activity inducing a VSMC dedifferentiated phenotype exhibiting procoagulant properties. An increase in FFAs contributes to the increased thrombin generation both in plasma and at the surface of VSMCs. Plasma from MSZR and palmitic acid elicit additive procoagulant effects. The potential benefit of direct thrombin inhibitors should be investigated both on haemostatic balance in blood compartments and on the cellular phenotypic modulation within the vessel wall, and MMP production in MetS and its complications with aging.

## Author contributions

JL: performed experiments, analyzed data and wrote the manuscript; AM, MD, and HL: performed experiments and analyzed data; LW, SB, and RA: contributed to the collection, analysis and interpretation of data and writing of the manuscript; BdL, BD, MS, ST, BF, PC, and JKC: contributed to critical writing and revising the intellectual content and final approval of the version of manuscript; PL and VR: designed research and supervised the work, analyzed the data, wrote and reviewed the manuscript.

### Conflict of interest statement

The authors declare that the research was conducted in the absence of any commercial or financial relationships that could be construed as a potential conflict of interest.

## References

[B1] Ait Aissa K. Lagrange J. Mohamadi A. Louis H. Houppert B. Challande P.. (2015). Vascular smooth muscle cells are responsible for a prothrombotic phenotype of spontaneously hypertensive rat arteries. Arterioscler. Thromb. Vasc. Biol. 35, 930–937. 10.1161/ATVBAHA.115.305377 25722431

[B2] Alessi M.-C. Juhan-Vague I. (2008). Metabolic syndrome, haemostasis, and thrombosis. Thromb. Haemost. 99, 995–1000. 10.1160/TH07-11-0682 18521499

[B3] Begbie M. Notley C. Tinlin S. Sawyer L. Lillicrap D. (2000). The Factor VIII acute phase response requires the participation of NFκB and C/EBP. Thromb. Haemost. 84, 216–222. 10959692

[B4] Beijers H. J. Ferreira I. Spronk H. M. Bravenboer B. Dekker J. M. Nijpels G.. (2010). Body composition as determinant of thrombin generation in plasma: the Hoorn study. Arterioscler. Thromb. Vasc. Biol. 30, 2639–2647. 10.1161/ATVBAHA.110.211946 20847307

[B5] Bendeck M. P. Irvin C. Reidy M. Smith L. Mulholland D. Horton M. (2000). Smooth muscle cell matrix metalloproteinase production is stimulated via α_v_β_3_ integrin. Arterioscler. Thromb. Vasc. Biol. 20, 1467–1472. 10.1161/01.ATV.20.6.1467 10845859

[B6] Boden G. Song W. Pashko L. Kresge K. (2008). *In vivo* effects of insulin and free fatty acids on matrix metalloproteinases in rat aorta. Diabetes 57, 476–483. 10.2337/db07-1261 18025411

[B7] Bourin M. C. (1987). [Effect of rabbit thrombomodulin on the inhibition of thrombin by the antithrombin-heparin complex: role of the acid domain of thrombomodulin]. Comptes Rendus Académie Sci. Sér. III Sci. Vie 304, 173–176. 3028582

[B8] Cesari M. Pahor M. Incalzi R. A. (2010). Plasminogen activator inhibitor-1 (PAI-1): a key factor linking fibrinolysis and age-related subclinical and clinical conditions. Cardiovasc. Ther. 28, e72–e91. 10.1111/j.1755-5922.2010.00171.x 20626406 PMC2958211

[B9] Chen Y.-J. Zhang L.-Q. Wang G.-P. Zeng H. Lü B. Shen X.-L.. (2008). Adiponectin inhibits tissue factor expression and enhances tissue factor pathway inhibitor expression in human endothelial cells. Thromb. Haemost. 100, 291–300. 10.1160/TH08-02-0124 18690350

[B10] Dandona P. Aljada A. Chaudhuri A. Mohanty P. Garg R. (2005). Metabolic syndrome: a comprehensive perspective based on interactions between obesity, diabetes, and inflammation. Circulation 111, 1448–1454. 10.1161/01.CIR.0000158483.13093.9D 15781756

[B11] Dinarello C. A. (2011). Interleukin-1 in the pathogenesis and treatment of inflammatory diseases. Blood 117, 3720–3732. 10.1182/blood-2010-07-273417 21304099 PMC3083294

[B12] Firszt R. Francisco D. Church T. D. Thomas J. M. Ingram J. L. Kraft M. (2014). Interleukin-13 induces collagen type-1 expression through matrix metalloproteinase-2 and transforming growth factor-β1 in airway fibroblasts in asthma. Eur. Respir. J. 43, 464–473. 10.1183/09031936.00068712 23682108 PMC6747688

[B13] Gabriel D. A. Muga K. Boothroyd E. M. (1992). The effect of fibrin structure on fibrinolysis. J. Biol. Chem. 267, 24259–24263. 1447176

[B14] Godin J.-P. Ross A. B. Cléroux M. Pouteau E. Montoliu I. Moser M.. (2013). Natural carbon isotope abundance of plasma metabolites and liver tissue differs between diabetic and non-diabetic Zucker diabetic fatty rats. PLoS ONE 8:e74866. 10.1371/journal.pone.0074866 24086387 PMC3781116

[B15] Halcox J. P. J. Donald A. E. Ellins E. Witte D. R. Shipley M. J. Brunner E. J.. (2009). Endothelial function predicts progression of carotid intima-media thickness. Circulation 119, 1005–1012. 10.1161/CIRCULATIONAHA.108.765701 19204308

[B16] Hotary K. B. Yana I. Sabeh F. Li X.-Y. Holmbeck K. Birkedal-Hansen H.. (2002). Matrix metalloproteinases (MMPs) regulate fibrin-invasive activity via MT1-MMP-dependent and -independent processes. J. Exp. Med. 195, 295–308. 10.1084/jem.20010815 11828004 PMC2193588

[B17] Konings J. Govers-Riemslag J. W. P. Philippou H. Mutch N. J. Borissoff J. I. Allan P.. (2011). Factor XIIa regulates the structure of the fibrin clot independently of thrombin generation through direct interaction with fibrin. Blood 118, 3942–3951. 10.1182/blood-2011-03-339572 21828145

[B18] Konstantinides S. Schäfer K. Koschnick S. Loskutoff D. J. (2001). Leptin-dependent platelet aggregation and arterial thrombosis suggests a mechanism for atherothrombotic disease in obesity. J. Clin. Invest. 108, 1533–1540. 10.1172/JCI13143 11714745 PMC209418

[B19] Kotronen A. Joutsi-Korhonen L. Sevastianova K. Bergholm R. Hakkarainen A. Pietiläinen K. H.. (2011). Increased coagulation factor VIII, IX, XI and XII activities in non-alcoholic fatty liver disease. Liver Int. 31, 176–183. 10.1111/j.1478-3231.2010.02375.x 21134109

[B20] Kumar R. Béguin S. Hemker H. C. (1994). The influence of fibrinogen and fibrin on thrombin generation–evidence for feedback activation of the clotting system by clot bound thrombin. Thromb. Haemost. 72, 713–721. 7900079

[B21] Lacolley P. Regnault V. Nicoletti A. Li Z. Michel J.-B. (2012). The vascular smooth muscle cell in arterial pathology: a cell that can take on multiple roles. Cardiovasc. Res. 95, 194–204. 10.1093/cvr/cvs135 22467316

[B22] Lee S. Park Y. Dellsperger K. C. Zhang C. (2011). Exercise training improves endothelial function via adiponectin-dependent and independent pathways in type 2 diabetic mice. Am. J. Physiol. Heart Circ. Physiol. 301, H306–H314. 10.1152/ajpheart.01306.2010 21602470 PMC3154670

[B23] Li Z. Froehlich J. Galis Z. S. Lakatta E. G. (1999). Increased expression of matrix metalloproteinase-2 in the thickened intima of aged rats. Hypertension 33, 116–123. 10.1161/01.HYP.33.1.116 9931091

[B24] Libby P. Ordovas J. M. Auger K. R. Robbins A. H. Birinyi L. K. Dinarello C. A. (1986). Endotoxin and tumor necrosis factor induce interleukin-1 gene expression in adult human vascular endothelial cells. Am. J. Pathol. 124, 179–185. 3526909 PMC1888305

[B25] Lim H. S. Lip G. Y. H. Blann A. D. (2004). Plasma von Willebrand factor and the development of the metabolic syndrome in patients with hypertension. J. Clin. Endocrinol. Metab. 89, 5377–5381. 10.1210/jc.2004-0616 15531484

[B26] Lominadze D. Dean W. L. Tyagi S. C. Roberts A. M. (2010). Mechanisms of fibrinogen-induced microvascular dysfunction during cardiovascular disease. Acta Physiol. Oxf. Engl. 198, 1–13. 10.1111/j.1748-1716.2009.02037.x 19723026 PMC2803614

[B27] Lu P. Liu J. Liu N. Guo F. Ji Y. Pang X. (2011). Pro-inflammatory effect of fibrinogen and FDP on vascular smooth muscle cells by IL-6, TNF-α and iNOS. Life Sci. 88, 839–845. 10.1016/j.lfs.2011.03.003 21439977

[B28] Mao X. Said R. Louis H. Max J.-P. Bourhim M. Challande P.. (2012). Cyclic stretch-induced thrombin generation by rat vascular smooth muscle cells is mediated by the integrin α_v_β_3_ pathway. Cardiovasc. Res. 96, 513–523. 10.1093/cvr/cvs274 22915765

[B29] Matsuzawa Y. Funahashi T. Kihara S. Shimomura I. (2004). Adiponectin and metabolic syndrome. Arterioscler. Thromb. Vasc. Biol. 24, 29–33. 10.1161/01.ATV.0000099786.99623.EF 14551151

[B30] Membre A. Wahl D. Latger-Cannard V. Max J.-P. Lacolley P. Lecompte T.. (2008). The effect of platelet activation on the hypercoagulability induced by murine monoclonal antiphospholipid antibodies. Haematologica 93, 566–573. 10.3324/haematol.12364 18322249

[B31] Mook O. R. F. Van Overbeek C. Ackema E. G. Van Maldegem F. Frederiks W. M. (2003). *In situ* localization of gelatinolytic activity in the extracellular matrix of metastases of colon cancer in rat liver using quenched fluorogenic DQ-gelatin. J. Histochem. Cytochem. 51, 821–829. 10.1177/002215540305100613 12754293

[B32] Morozumi T. Sharma A. De Nardin E. (2009). The functional effects of the−455G/A polymorphism on the IL-6-induced expression of the beta-fibrinogen gene may be due to linkage disequilibrium with other functional polymorphisms. Immunol. Invest. 38, 311–323. 10.1080/08820130902745153 19811441

[B33] Ninivaggi M. Apitz-Castro R. Dargaud Y. de Laat B. Hemker H. C. Lindhout T. (2012). Whole-blood thrombin generation monitored with a calibrated automated thrombogram-based assay. Clin. Chem. 58, 1252–1259. 10.1373/clinchem.2012.184077 22665918

[B34] Pacheco Y. M. Bermúdez B. López S. Abia R. Villar J. Muriana F. J. G. (2006). Ratio of oleic to palmitic acid is a dietary determinant of thrombogenic and fibrinolytic factors during the postprandial state in men. Am. J. Clin. Nutr. 84, 342–349. 16895881 10.1093/ajcn/84.1.342

[B35] Pandolfi A. Cetrullo D. Polishuck R. Alberta M. M. Calafiore A. Pellegrini G.. (2001). Plasminogen activator inhibitor type 1 is increased in the arterial wall of type II diabetic subjects. Arterioscler. Thromb. Vasc. Biol. 21, 1378–1382. 10.1161/hq0801.093667 11498469

[B36] Paul W. Queen L. R. Page C. P. Ferro A. (2007). Increased platelet aggregation *in vivo* in the Zucker Diabetic Fatty rat: differences from the streptozotocin diabetic rat. Br. J. Pharmacol. 150, 105–111. 10.1038/sj.bjp.0706957 17099716 PMC2013856

[B37] Petrini S. Neri T. Lombardi S. Cordazzo C. Balìa C. Scalise V.. (2016). Leptin induces the generation of procoagulant, tissue factor bearing microparticles by human peripheral blood mononuclear cells. Biochim. Biophys. Acta 1860, 1354–1361. 10.1016/j.bbagen.2016.03.029 27015759

[B38] Phillips M. S. Liu Q. Hammond H. A. Dugan V. Hey P. J. Caskey C. J.. (1996). Leptin receptor missense mutation in the fatty Zucker rat. Nat. Genet. 13, 18–19. 10.1038/ng0596-18 8673096

[B39] Regnault V. Hemker H. C. Wahl D. Lecompte T. (2004). Phenotyping the haemostatic system by thrombography–potential for the estimation of thrombotic risk. Thromb. Res. 114, 539–545. 10.1016/j.thromres.2004.06.017 15507289

[B40] Restituto P. Colina I. Varo J. J. Varo N. (2010). Adiponectin diminishes platelet aggregation and sCD40L release. Potential role in the metabolic syndrome. Am. J. Physiol. Endocrinol. Metab. 298, E1072–E1077. 10.1152/ajpendo.00728.2009 20197504

[B41] Ridker P. M. Everett B. M. Thuren T. MacFadyen J. G. Chang W. H. Ballantyne C.. (2017). Antiinflammatory therapy with canakinumab for atherosclerotic disease. N. Engl. J. Med. 377, 1119–1131. 10.1056/NEJMoa1707914 28845751

[B42] Samad F. Pandey M. Loskutoff D. J. (2001). Regulation of tissue factor gene expression in obesity. Blood 98, 3353–3358. 10.1182/blood.V98.12.3353 11719374

[B43] Semeraro F. Ammollo C. T. Morrissey J. H. Dale G. L. Friese P. Esmon N. L.. (2011). Extracellular histones promote thrombin generation through platelet-dependent mechanisms: involvement of platelet TLR2 and TLR4. Blood 118, 1952–1961. 10.1182/blood-2011-03-343061 21673343 PMC3158722

[B44] Shang J. Chen Z. Wang M. Li Q. Feng W. Wu Y.. (2014). Zucker diabetic fatty rats exhibit hypercoagulability and accelerated thrombus formation in the Arterio-Venous shunt model of thrombosis. Thromb. Res. 134, 433–439. 10.1016/j.thromres.2014.04.008 24796819

[B45] Shrestha C. Ito T. Kawahara K. Shrestha B. Yamakuchi M. Hashiguchi T.. (2013). Saturated fatty acid palmitate induces extracellular release of histone H3: a possible mechanistic basis for high-fat diet-induced inflammation and thrombosis. Biochem. Biophys. Res. Commun. 437, 573–578. 10.1016/j.bbrc.2013.06.117 23850687

[B46] Simon J. A. Hodgkins M. L. Browner W. S. Neuhaus J. M. Bernert J. T. Hulley S. B. (1995). Serum fatty acids and the risk of coronary heart disease. Am. J. Epidemiol. 142, 469–476. 10.1093/oxfordjournals.aje.a117662 7677125

[B47] Sloboda N. Fève B. Thornton S. N. Nzietchueng R. Regnault V. Simon G.. (2012). Fatty acids impair endothelium-dependent vasorelaxation: a link between obesity and arterial stiffness in very old Zucker rats. J. Gerontol. A Biol. Sci. Med. Sci. 67, 927–938. 10.1093/gerona/glr236 22389459

[B48] Sonnenberg G. E. Krakower G. R. Kissebah A. H. (2004). A novel pathway to the manifestations of metabolic syndrome. Obes. Res. 12, 180–186. 10.1038/oby.2004.24 14981209

[B49] Suehiro A. Wakabayashi I. Uchida K. Yamashita T. Yamamoto J. (2012). Impaired spontaneous thrombolytic activity measured by global thrombosis test in males with metabolic syndrome. Thromb. Res. 129, 499–501. 10.1016/j.thromres.2011.06.019 21752433

[B50] Wagenvoord R. J. Hendrix H. H. Kai H. Hemker H. C. (1994). A chromogenic test to determine the procoagulant phospholipids in platelet-rich plasma and whole blood. Thromb. Haemost. 72, 582–587. 7878637

[B51] Wakil S. J. Abu-Elheiga L. A. (2009). Fatty acid metabolism: target for metabolic syndrome. J. Lipid Res. 50, S138–S143. 10.1194/jlr.R800079-JLR200 19047759 PMC2674721

[B52] Wang M. Y. Zhou Y. T. Newgard C. B. Unger R. H. (1996). A novel leptin receptor isoform in rat. FEBS Lett. 392, 87–90. 10.1016/0014-5793(96)00790-9 8772180

[B53] Wang M. Zhang J. Jiang L.-Q. Spinetti G. Pintus G. Monticone R.. (2007). Proinflammatory profile within the grossly normal aged human aortic wall. Hypertension 50, 219–227. 10.1161/HYPERTENSIONAHA.107.089409 17452499

[B54] Wanninger J. Walter R. Bauer S. Eisinger K. Schäffler A. Dorn C.. (2011). MMP-9 activity is increased by adiponectin in primary human hepatocytes but even negatively correlates with serum adiponectin in a rodent model of non-alcoholic steatohepatitis. Exp. Mol. Pathol. 91, 603–607. 10.1016/j.yexmp.2011.07.001 21791204

[B55] Weiss T. W. Arnesen H. Seljeflot I. (2013). Components of the interleukin-6 transsignalling system are associated with the metabolic syndrome, endothelial dysfunction and arterial stiffness. Metab. Clin. Exp. 62, 1008–1013. 10.1016/j.metabol.2013.01.019 23428306

[B56] Wolberg A. S. (2007). Thrombin generation and fibrin clot structure. Blood Rev. 21, 131–142. 10.1016/j.blre.2006.11.001 17208341

[B57] Zhou Y.-P. Madjidi A. Wilson M. E. Nothhelfer D. A. Johnson J. H. Palma J. F.. (2005). Matrix metalloproteinases contribute to insulin insufficiency in Zucker diabetic fatty rats. Diabetes 54, 2612–2619. 10.2337/diabetes.54.9.2612 16123349

